# Dynamic changes in mitochondrial 3D structure during folliculogenesis and luteal formation in the goat large luteal cell lineage

**DOI:** 10.1038/s41598-021-95161-w

**Published:** 2021-07-30

**Authors:** Yi-Fan Jiang, Pin-Huan Yu, Yovita Permata Budi, Chih-Hsien Chiu, Chi-Yu Fu

**Affiliations:** 1 Graduate Institute of Molecular and Comparative Pathobiology, School of Veterinary Medicine, National Taiwan University, Rm. 104-1, No.1, Sec. 4, Roosevelt Road, Taipei City, 10617 Taiwan, ROC; 2 Institute of Veterinary Clinical Science, School of Veterinary Medicine, National Taiwan University, Taipei, Taiwan, ROC; 3 Department of Animal Science and Technology, National Taiwan University, Taipei, Taiwan, ROC; 4 Institute of Cellular and Organismic Biology, Academia Sinica, Taipei, Taiwan, ROC

**Keywords:** Biological techniques, Biophysics, Cell biology, Developmental biology, Physiology, Endocrinology

## Abstract

In mammalian ovaries, mitochondria are integral sites of energy production and steroidogenesis. While shifts in cellular activities and steroidogenesis are well characterized during the differentiation of large luteal cells in folliculogenesis and luteal formation, mitochondrial dynamics during this process have not been previously evaluated. In this study, we collected ovaries containing primordial follicles, mature follicles, corpus hemorrhagicum, or corpus luteum from goats at specific times in the estrous cycle. Enzyme histochemistry, ultrastructural observations, and 3D structural analysis of serial sections of mitochondria revealed that branched mitochondrial networks were predominant in follicles, while spherical and tubular mitochondria were typical in large luteal cells. Furthermore, the average mitochondrial diameter and volume increased from folliculogenesis to luteal formation. In primordial follicles, the signals of cytochrome c oxidase and ATP synthase were undetectable in most cells, and the large luteal cells from the corpus hemorrhagicum also showed low enzyme signals and content when compared with granulosa cells in mature follicles or large luteal cells from the corpus luteum. Our findings suggest that the mitochondrial enlargement could be an event during folliculogenesis and luteal formation, while the modulation of mitochondrial morphology and respiratory enzyme expressions may be related to tissue remodeling during luteal formation.

## Introduction

The development of follicles and formation of corpus luteum (CL) are the major processes that define the two phases of the ovarian cycle. During these phases, endocrine function of the ovary and support for oocyte development rely heavily on the differentiation of the large luteal cell (LLC) lineage. Development of this lineage begins when follicle cells (FCs), which reside in primordial follicles (PFs), proliferate and stratify into granulosa cells (GCs) during folliculogenesis. As a dominant mature follicle (MF) escapes from follicular atresia, estrogen production by a maximal number of GCs triggers a surge of luteinizing hormone (LH) to stimulate ovulation. Shortly after ovulation, the GCs differentiate into LLCs during maturation of the corpus hemorrhagicum (CH) into the CL. Secretion of progesterone (P_4_) from the CL then provides necessary support for development of an embryo^1^.

Mitochondria are structurally unique organelles that serve not only as the power plant of the cell but also as a signaling platform in cell physiology. Structurally, mitochondria are organelles with double membranes called the outer mitochondrial membrane (OMM) and the inner mitochondrial membrane (IMM). The OMM separates the organelle from the cytoplasm, while the IMM divides the organelle into intermembrane space and matrix compartments. Invagination of the IMM into the matrix creates cristae, which house respiratory protein complexes (complexes I–IV) for ATP synthesis^2^. Mitochondrial ATP production requires the consumption of intracellular oxygen to support cellular functions and cell survival, but a toxic byproduct of mitochondrial oxygen consumption is the generation of reactive oxygen species (ROS). Imbalances in mitochondrial ATP and ROS production may lead to cell death via opening of the mitochondrial permeability transition pore on the surface of mitochondria and subsequent activation of apoptosis^3^. In post-ovulation ovarian tissues, the CH undergoes dramatic tissue remodeling with rapid proliferation and differentiation of GCs, and during this time, signs of hypoxia and angiogenesis can be observed^4^. Since ischemia and reperfusion is known to cause mitochondrial dysfunction and cellular damage, differentiating GCs may recruit certain adaptive mechanisms to survive in the microenvironment. Besides their role in energy production, mitochondria in cells of the LLC lineage also provide a regulatory site for steroidogenesis; the rate-limiting steps of P_4_ production are mitochondrial cholesterol transport and a side-chain cleavage reaction by the mitochondrial enzyme CYP11A1^5^. However, specific roles for mitochondria in survival of LLCs within the CH or steroidogenesis (P_4_ production) by LLCs in the CL have not been defined.

Since the content and quality of mitochondria can be regulated by mitochondrial dynamics (fission and fusion), biogenesis and mitophagy, the morphology of mitochondrial networks may reflect various roles played by the organelle at specific stages of cell differentiation^6^. In the present study, we sampled goat ovaries based on estrous cycle prediction and investigated the structural and functional changes of mitochondria in the LLC lineage during follicle-luteal transitions. To examine fine structural details, 3D reconstructions of cellular structures and enzyme activities were made at an ultrastructural level.

## Results

### Cycle prediction and microscopic cell identification

**Figure 1:**
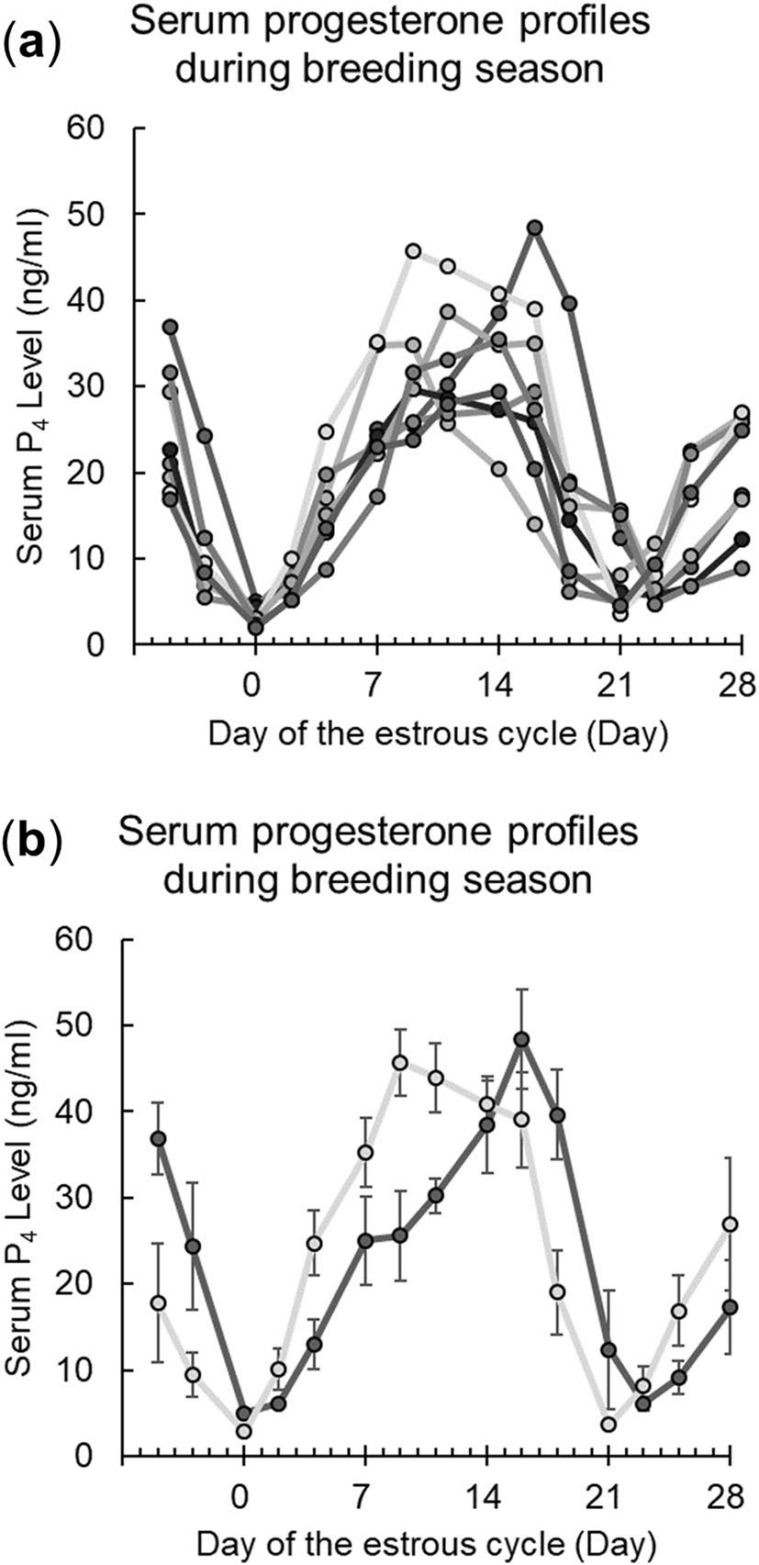
Goat serum progesterone profiles are shown. (**a**) The representative P_4_ profiles of the participant animals (n = 8) showed cycle lengths of 21–23 days. For each individual, the representative curve was averaged from 4 to 7 cycles. (**b**) two representative curves from (**a**) (21-day and 23-day cycle) are shown with the variation (Mean ± SEM).

**Figure 2:**
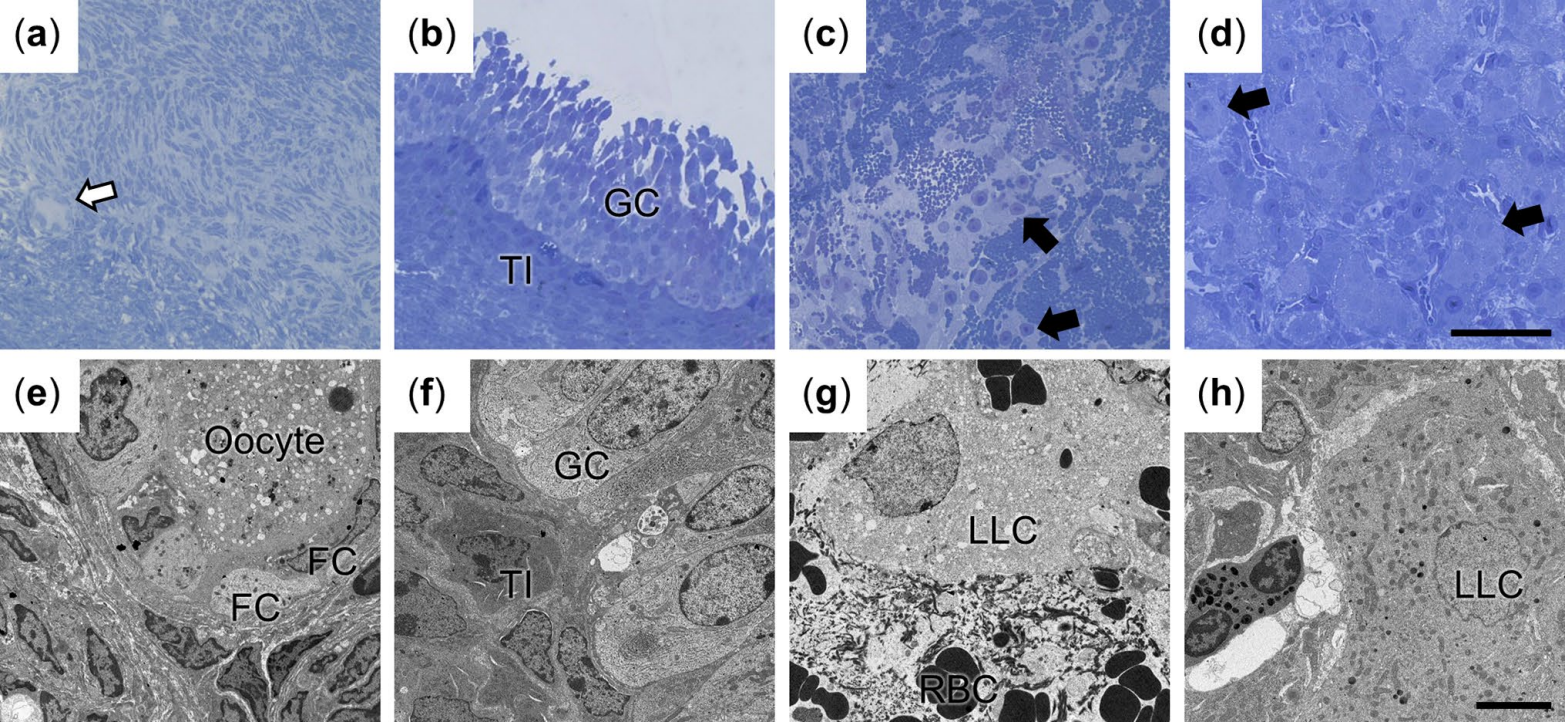
Representative images show the lineage of LLCs in goat ovarian tissues at a cellular level. (**a**–**d**) The images depict PF (white arrow, **a**), MF (**b**), CH (**c**) and CL (**d**). The black arrow indicates LLCs. Bar: 100 μm. (**e**–**h**) The TEM images show FCs (**e**), GCs (**f**), LLCs in CH (**g**) and LLCs in CL (**h**) in corresponding tissues at low magnifications. *TI* theca interna cells, *RBC* red blood cells. Bar: 5 μm.

To obtain cells from the goat LLC lineage at various developmental stages, serum progesterone profiles were monitored for cycle prediction and sampling (Fig. 1). Although the cycle length of individual does varied from 21 to 23 days in our study (Fig. 1a), the cycle lengths for each animal were consistent and predictable (Fig. 1b). Ovarian tissues were sampled based the predicted day of ovulation, and tissues with PFs, MFs, CHs, and CLs were successfully obtained (Fig. 2). Since TEM revealed variable cellular characteristics, we focused our structural investigations on the squamous to cuboidal FCs in PFs (Fig. 2a,e), the basal columnar GCs in MF (Fig. 2b,f), the LLCs with large cell size (~ 20 μm) in CH (Fig. 2c,g) and CL (Fig. 2d,h).

### 2D mitochondrial structures in goat LLC lineage

**Figure 3:**
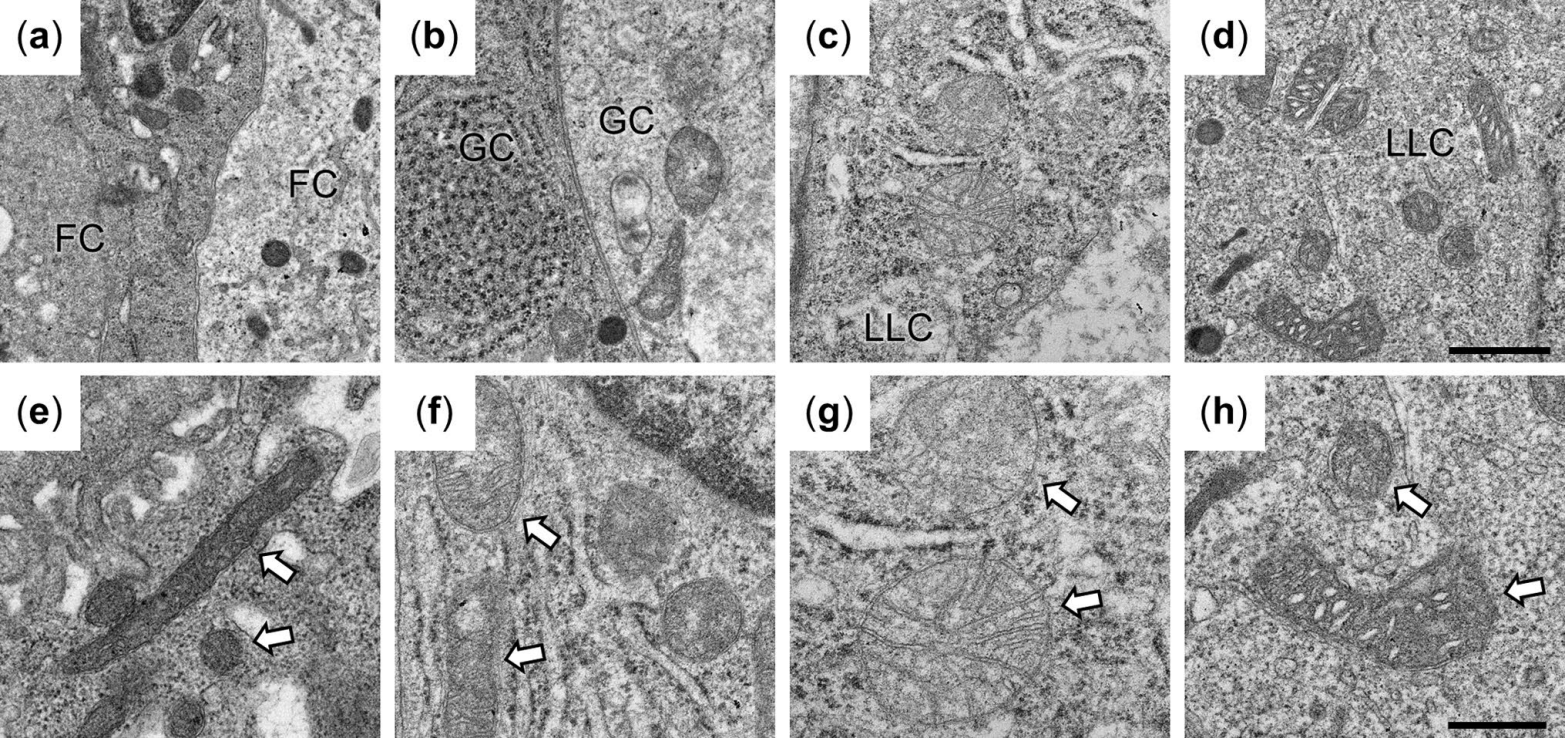
The TEM images show distributions of mitochondria (**a**–**d**) and cristae structures (**e**–**h**) in goat FC (**a**, **e**), GC (**b**, **f**), LLCs in CH (**c**, **g**) and LLCs in CL (**d**, **h**). The white arrows indicate mitochondria shown at high magnification. Bar: 1 μm (**a**–**d**) and 0.5 μm (**e**–**h**).

To assess mitochondrial structure, 2D cellular images of goat LLC lineage were collected at higher magnifications (Fig. 3). In the cytoplasm of PFs, small round- and rod-shaped mitochondria could be found (Fig. 3a,e). Although the double membrane was discernable in our specimens, no highly organized cristae were observed (Fig. 3e). In GCs, the mitochondrial shapes were similar to those in PFs (Fig. 3b,f). However, the connectivity of organelles could not be properly evaluated from the 2D images. In mitochondria, both pale- or dark-staining matrix were found. The cristae may form lamellar structures, which are observed as parallel straight lines in the organelle (Fig. 3f). In the LLCs from CH, mitochondria were predominantly round-shaped with lamellar cristae and pale-staining matrix (Fig. 3c,g). Elongated mitochondria or rod-like shapes were less common in CH. The LLC mitochondria from CLs exhibited dark matrix and tubular cristae, with the cristae membrane arranged in circular structures in the cross-sections (Fig. 3d,h). Also, multiple shapes (round, rod-like, or bean-like) of mitochondria were observed in 2D sections of CLs (Fig. 3h).

### Morphological 3D analysis

**Figure 4:**
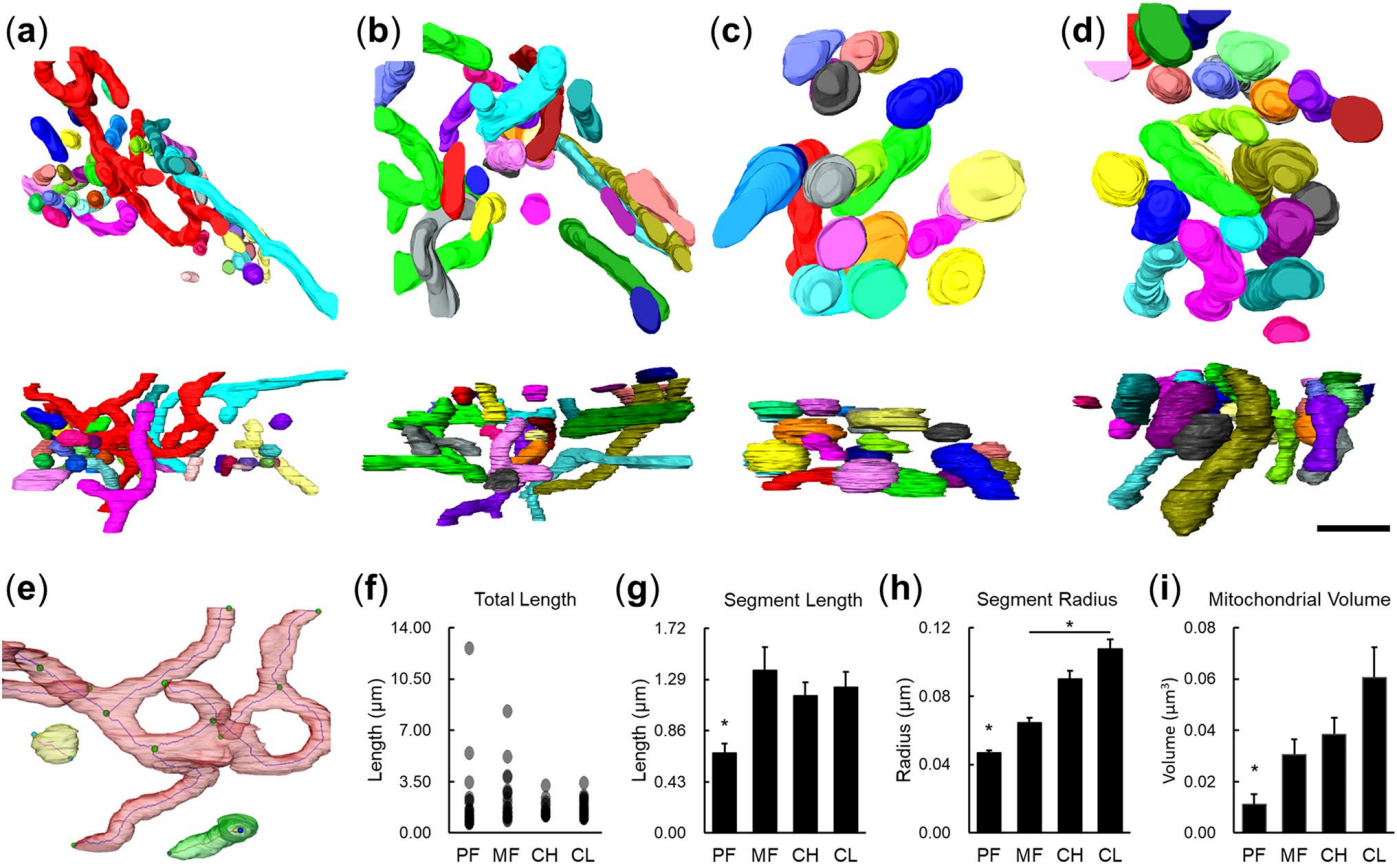
Visualization and quantitative analysis of 3D mitochondrial networks in the goat LLC lineage. (**a**–**d**) The top view (the upper panel) and side view (the lower panel) of mitochondrial networks in FC (**a**), GC (**b**), LLC in CH (**c**) and LLC in CL (**d**). Mitochondria without physical connections are labeled with different colors. Bar: 1 μm. (**e**) The diagram shows nodes (colored spheres) and central line segments (the line between two nodes) of mitochondria (labeled in transparent red color) in FC. (**f**) The distributions of total length of the central line segments in mitochondria from the LLC lineage. (**g**, **h**) The average of length (**g**) and mean radius (**h**) of the mitochondrial segments in the LLC lineage. (**i**) The average volumes of mitochondria in the LLC lineage. Data are presented as the mean ± SEM (n = 39 for PF, 22 for MF, 21 for CH, and 24 for CL). Statistical analysis was done with one-way ANOVA and Dunn’s multiple comparison test. *P < 0.05.

To visualize the mitochondrial network during development of the goat LLC lineage, serial sections and electron tomography were performed (Fig. 4 and Supplemental Movies 1, 2, 3 and 4). To evaluate the sizes and connectivity of the organelles, small mitochondria without clearly defined volumes were excluded. The top views and side views of selected mitochondria after manual segmentation are displayed in Fig. 4a–d. After segmentation, the morphologies of mitochondrial networks were analyzed according to the segments and nodes on the central line tree (Fig. 4e). In FCs and GCs, several branched and long (total length) mitochondrial networks were observed (Fig. 4a,b,d; Supplemental Movie 1 for FC and Supplemental Movie 2 for GC), while mitochondrial branching was absent in the LLCs from CH (Supplemental Movie 3) or CL (Supplemental Movie 4). Nearby the branched networks in PFs and MFs, smaller mitochondria could be observed (Fig. 4a,b). Since the data did not follow a normal distribution, a distribution-free test was applied for statistical analysis. On average, the smallest mitochondrial segment length, radius and volume were all observed in FCs (Fig. 4g–i). The volume and radius of mitochondria seemed to be increased gradually from PF to CL stages, and significant differences were found between the mitochondrial radii in MFs and CL (Fig. 4h).

### The metabolic status and cristae structures of mitochondria

**Figure 5:**
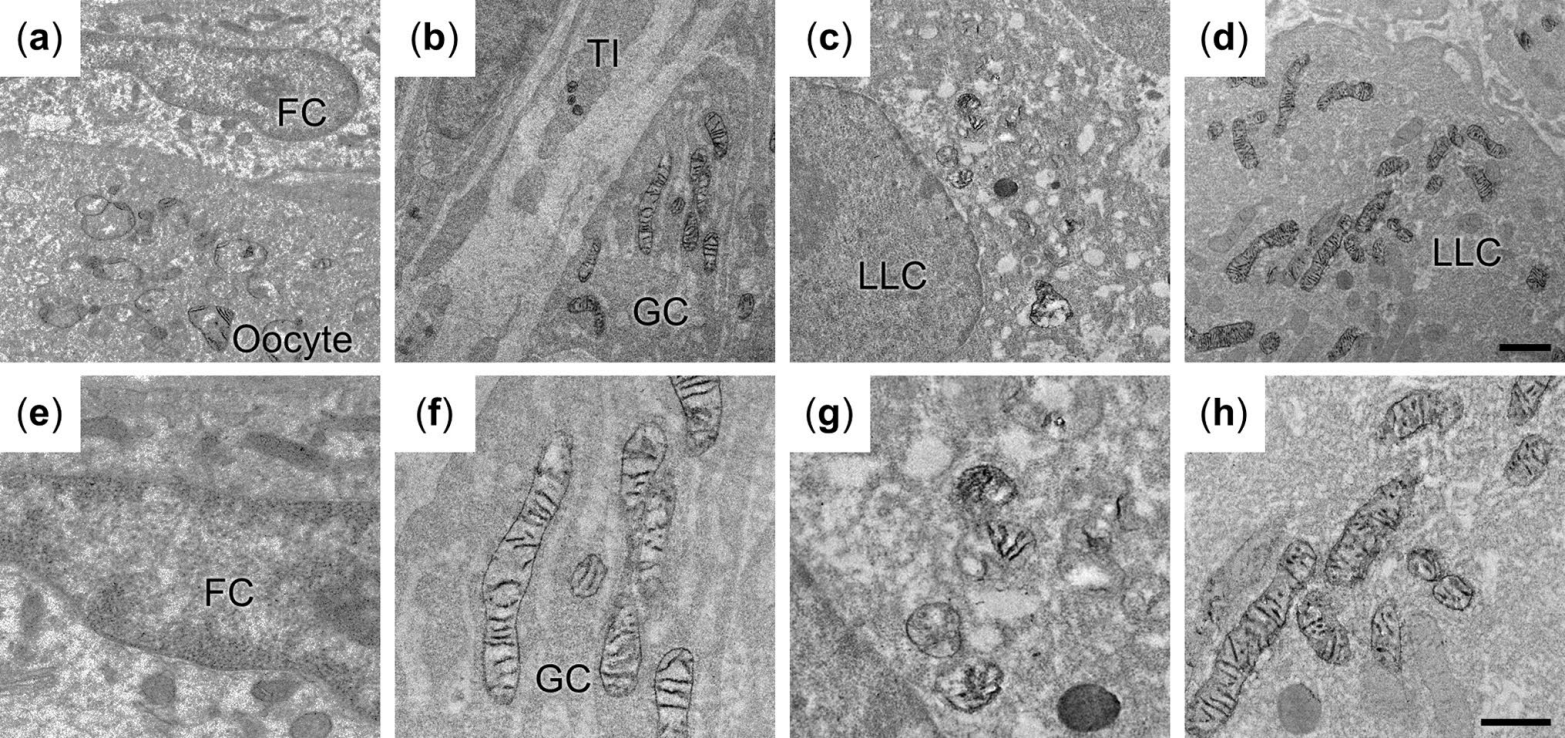
The distributions of COX signals in the goat LLC lineage. Images of COX signals (dark densities) were obtained at low (**a**–**d**) and high magnifications (**e**–**h**) in FC (**a**, **e**), GC (**b**, **f**), LLC in CH (**c**, **g**) and LLC in CL (**d**, **h**). *TI* theca interna cell. Bar: 1 μm (**a**–**d**) and 0.5 μm (**e**–**h**).

Since the cristae are the site of many mitochondrial enzyme reactions, we performed a histochemical stain for COX activity to probe the relationship between structure of cristae and the distribution of respiratory function (Fig. 5). The COX signals were observed at the IMS and the cristae (either lamellar or tubular types) in our study. Although the oocytes showed strong COX signals on cristae of PFs, most FCs showed weak or undetectable COX signals under TEM (Fig. 5a,e). In GCs and LLCs, both COX-positive (strong signal) and COX-negative (weak signal) mitochondria were observed (Fig. 5b-d,f–h).

**Figure 6:**
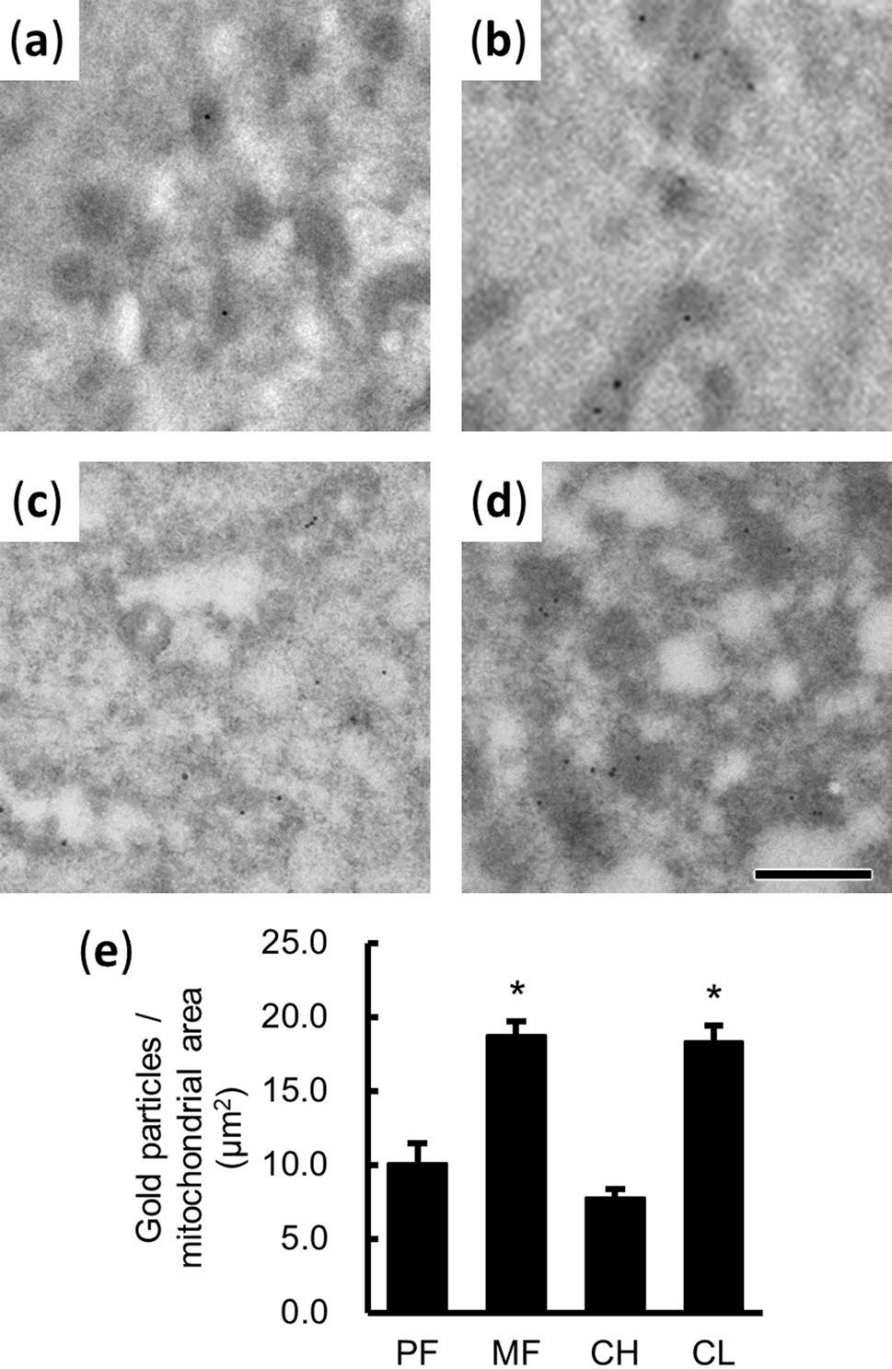
The expression of ATP synthase (ATP5A) on the lineage of LLCs in goat ovarian tissues. (**a**–**d**) Representative images of the immuno-gold labeling of ATP5A in goat FC (**a**), GC (**b**), LLCs in CH (**c**), and LLCs in CL (**d**). Bar = 0.5 μm. (**e**) The 2D quantitative analysis of gold particles on discernable mitochondrial areas. Data are presented as the mean ± SEM (the analyzed mitochondria: n = 20 for PF, 121 for MF, 38 for CH, and 98 for CL). Statistical analysis was done with one-way ANOVA and Dunn’s multiple comparison test. *P < 0.05 when compared to PF or CH groups.

To confirm the distribution and expression of mitochondria respiratory complexes, immuno-gold labeling of ATP synthase (ATP5A) was performed on the ultrathin section containing the cells of LLC lineage (Fig. 6a–d). Since better antigenicity but less structural information was preserved in the tissues without post-fixation, the mitochondria with a clear boundary and positive signals were chosen for quantitative analysis (Fig. 6e). The denser distributions of gold particles were found in the mitochondrial area of MF (granulosa cells) and CL (LLCs) when compared to those in PF (follicle cells) and CH (developing LLCs, Fig. 6e).

### 3D analysis of COX activities

**Figure 7:**
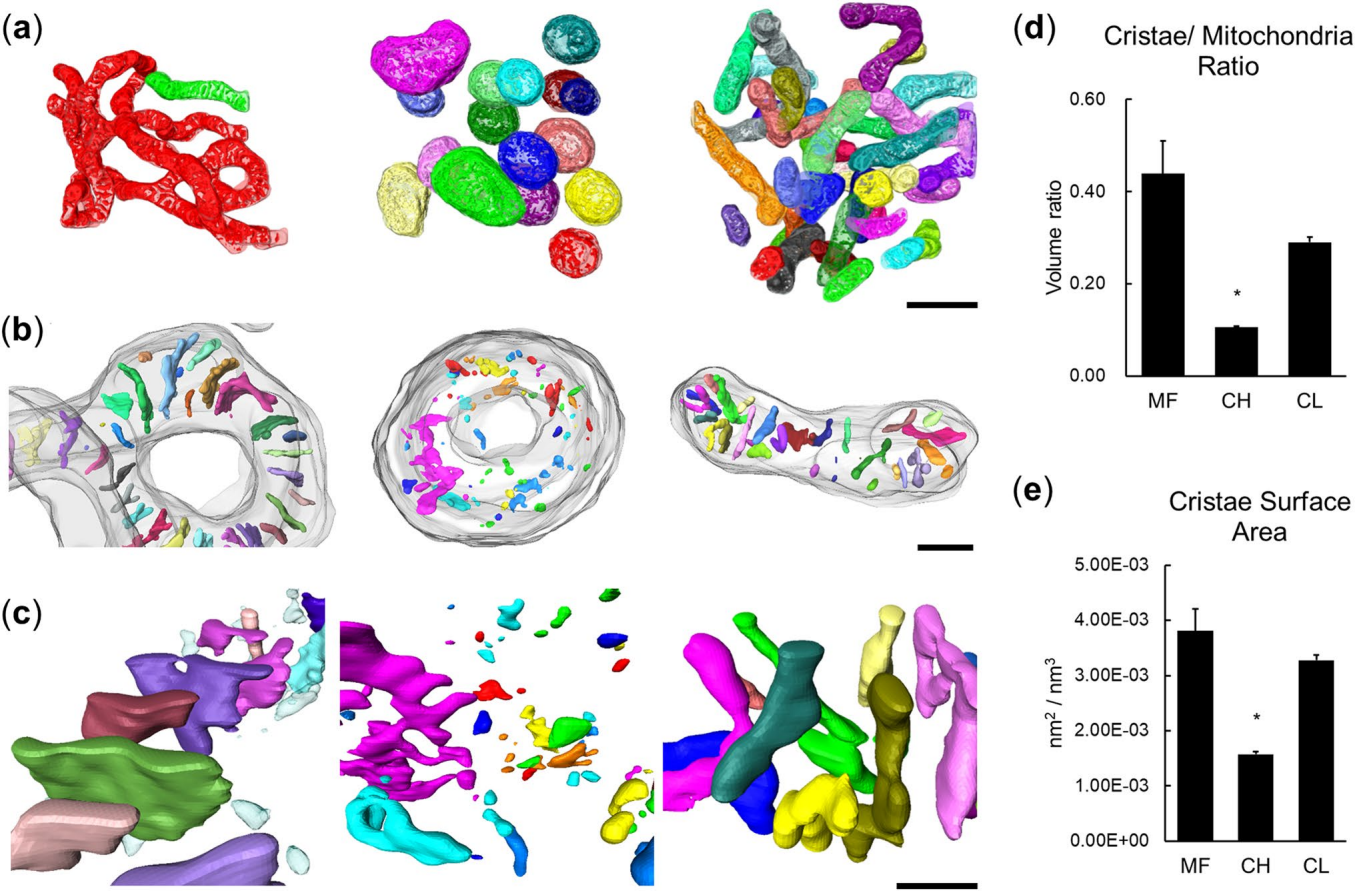
Visualization and quantitative analysis of mitochondrial networks with COX-positive signal in the goat LLC lineage. (**a**) Mitochondria with COX signals in GC (left), LLC in CH (middle) and LLC in CL (right). The mitochondria without physical connections are labeled with different colors. Bar: 1 μm. (**b**) Representative distributions of cristae COX signals (colored segments) in the mitochondria (gray) from GC (left), LLC in CH (middle), and LLC in CL (right). Bar: 0.2 μm. (**c**) The structure of cristae at higher magnification. Left: GC. Middle: LLC in CH. Right: LLC in CL. Bar: 0.1 μm. (**d**) The average volume ratio between the cristae and mitochondria in the LLC lineage. (**e**) The surface area of the cristae in the mitochondria from the LLC lineage. Data are presented as the mean ± SEM (the total COX positive mitochondria analyzed: n = 7 for MF, 17 for CH, and 37 for CL). Statistical analysis was performed with one-way ANOVA and the Dunn’s multiple comparison test. *P < 0.05.

Since the COX-positive mitochondria were rare in FCs (PF), we focused our 3D structural analysis on cells in MF (7 mitochondria from 2 data sets), CH (17 mitochondria from 1 data set), and CL (37 mitochondria from 1 data set; Fig. 7 and Supplemental Movies 5, 6 and 7). Interestingly, the COX-positive mitochondria formed branched mitochondrial networks in MFs (Fig. 7a and Supplemental Movie 5). Moreover, the distribution of COX signals formed lamellae in MF mitochondria and tubules in CL mitochondria (Fig. 7b,c; Supplemental Movies 5 and 7). Fragmented and scattered lamellar distributions were found in CH (Fig. 7b,c; Supplemental Movie 6). In our structural analysis of mitochondria with the cristae covered by COX signals, the smallest cristae volume and cristae surface area density were found in CH mitochondria (Fig. 7d,e).

## Discussion

Mitochondria are involved in apoptosis, ATP production and steroidogenesis at various stages of differentiation in the LLC linage. Therefore, biogenesis of the organelle is an important process for ovarian tissue homeostasis. PGC-1α is considered to be a master switch for mitochondrial biogenesis in mammalian cells, and recent studies in goat follicles have shown that the expression of PGC-1α serves as a survival factor for GCs^7,8^. Furthermore, overexpression of nuclear respiratory factor 1 (NRF1), which also mediates mitochondrial biogenesis, enhances COX activity and P_4_ production in goat luteinized GCs^9^. Based on these previous reports and our analysis at the level of single organelles, we conclude that mitochondrial biogenesis may explain the mitochondrial mass increases (radius and volume) we observed from PF to CL.

Although the switch for biogenesis of mitochondrial protein components has been highlighted in the literature, the biogenic regulations of mitochondrial lipids and membranes are still under investigation^10^. During the stage of CH, the increased mitochondrial radius and volume did not accompany more cristae structures, nor higher expression levels of COX and ATP synthase within the organelles. Our structural analysis suggested that the biogenesis of cristae (respiratory complexes) and mitochondrial volume could be regulated independently. While the electron tomography on serial sections can be applied to reconstruct the missing dimension of conventional 2D images at the ultrastructural level, the volume and scale of observation are still limited. In goat ovarian tissues, the diameter of LLCs could exceed 20 μm in 2D sections, while the optimized thickness of semi-thin sections for electron tomography was 200 nm in our system, suggesting more than 100 sections will be needed to assemble a whole-cell volume. Since the collection of continuous sections for whole-cell volume could still be difficult, other imaging methods might be necessary to compare the ratio of mitochondrial volume to whole-cell volume. Also, since the CHs and CLs are mixed tissues with multiple cell types, the establishment of in vitro models could be a more effective way to investigate the mitochondrial function and gene expressions in the developing LLC lineage.

In addition to changes in mass, significant changes in mitochondrial shape were noted during the MF-CL transition; long and branched networks in follicles transformed to spherical mitochondria in CH. From previous reports, mitochondrial morphology is known to respond to various stimuli. For example, uncoupled (depolarized) mitochondria shorten their long axis to form vase structures; such changes might include the expansion of the short axis and the indention of the spheroids^11^. Swollen mitochondria may have an open mitochondrial permeability transition pore, which would be expected to drive apoptosis in the cell^12^. In ischemia/reperfusion injury, it has been observed that the activities of COX could have an initial hyperactive state to compensate for the depletion of ATP in the ischemic starvation phase. And the COX activities could be dramatically inhibited if mitochondrial dysfunction (energy failure) and apoptosis occurred at the final phase of reperfusion injury^13^. However, the apoptosis rate in LLCs should be low during CL formation^14^. Since the CH mitochondria in our study showed intact IMM and OMM without structural indentions, the shift in mitochondrial morphology from MF to CH likely reflects a remodeling event related to cell differentiation.

Currently, little information about mitochondrial 3D structures in ovarian tissue is available. In stem cells, recent studies have made associations of mitochondrial shapes and activities with developmental potential and reprogramming, as branched and complex mitochondrial networks are often found in differentiated progenitor cells^6,15,16^. It was also suggested that the cells with elongated mitochondria have higher activities of oxidative phosphorylation, ROS, and ATP production^15^. This is consistent with our 3D networks of COX-positive mitochondria in MF, CH, and CL. While the smaller COX-negative mitochondria in round shapes could average the quantitative results in our morphological preparations, their biological significance is still unclear. The mitochondrial network could also be linked to cell cycles, where hyper-fused networks can be found in the G1-S phase with fragmented mitochondria observed in mitosis^16^. Although cell proliferation and differentiation were known in luteal formation, the round mitochondria in CH might not be totally identical to mitochondrial fragmentation. Also, our data suggest that FCs and basal GCs contain branched mitochondrial networks. However, since GCs could have heterogeneous cellular activities, in vitro or ex vivo study with mitochondrial dynamic modulators on ovarian tissue will be necessary to define the dynamics and roles of the mitochondrial network in folliculogenesis and luteal formation^17–19^.

To estimate the function of mitochondrial ATP production in the LLC lineages, immuno-gold labeling of ATP synthase and enzyme histochemistry stain of COX were applied in our study. As the enzyme histochemistry of COX could be accommodated into the TEM sample preparations for high-resolution imaging, the enzyme histochemistry of COX was also used to visualize the structure of cristae in this study. The distribution of COX signals was mainly limited to the intermembrane space and cristae. Tubular cristae in steroidogenic mitochondria have been reported in several tissues and species^20–22^. The activity of CYP11A1 is driven by electrons from NADPH, which may be produced via a pathway independent from ATP production. In our results, the distribution of COX signals in CL mitochondria revealed that cristae ATP production and steroidogenesis might be physically separated in tubular cristae. Interestingly, mitochondria with heterogeneous COX signals were typically observed within the cytoplasm of the same cells. Since the COX-negative mitochondria seemed to be randomly distributed among COX-positive organelles, the appearance of distinct mitochondrial subpopulations may not be an artifact of fixation and staining protocols. To investigate the roles of mitochondrial subpopulations on the estrous cycle and reproductive systems, better throughput of sampling and analysis might be necessary. Also, since the methods with other platforms (ex: fluorescence labeling, cell sorting, and colocalization analysis) could provide functional information and effective observations, the correlations among the analytic tools could also be necessary^23^.

The FCs in PFs very rarely contained COX- and ATP5A-positive mitochondria, suggesting the FCs might be considered as a ‘resting’ cell in follicles^24^. In line with this idea, the activation of respiratory ATP production has been suggested to be an initial cellular event in folliculogenesis^25,26^. Furthermore, a metabolic shift between glycolysis and mitochondrial oxidative phosphorylation has been noted in the differentiation and activation of many cell types under hypoxia^27,28^. Decreases in oxidative phosphorylation are often accompanied by decreased mitochondrial elongation (fusion) and increased mitochondrial fragmentation (fission)^29^. Since hypoxia may occur during luteal formation in vivo, our study suggests that the breakdown of mitochondrial networks and decrease in the distributions of COX and ATP synthase could reflect a decrease of loading on oxidative phosphorylation for each mitochondrion in the CH^30^. Having already completed luteal formation, the COX and cristae remodeling could reflect different cellular activities of the LLCs in the CL.

In conclusion, our observations suggest that mitochondrial enlargement may occur throughout follicle and early CL development, while the fission of mitochondria may be responsible for the later breakdown of mitochondrial networks. Our data also suggest that the respiratory function of mitochondria could be higher in MFs than in PFs. In spite of the diminished functional role of the LLC lineage in the CH, respiratory and steroidogenic cristae could still play a role in the CL. Overall, our observations set the stage for future studies that explore the impacts and regulatory mechanisms of mitochondrial dynamics in the ovarian cycle.

## Material and methods

### Animals

Adult Saanen and Alpine crossbreed does (n = 11) were housed at the Experimental Farm of National Taiwan University. Animals were maintained on natural dark–light cycles. Water and grass were available ad libitum. All experiments were conducted in accordance with the Guide for Care and Use of Laboratory Animals and were approved by the Research Ethics Office of National Taiwan University. All methods were also performed in accordance with ARRIVE guidelines. Blood samples were collected three times per week, and the concentrations of serum P_4_ were determined by an enzyme immunoassay (EIA) to predict estrous cycles^21^.

### Enzyme immunoassay for serum progesterone concentration

Serum P_4_ concentrations were determined using a competitive EIA, as previously described^31^. All standards and samples were assayed in duplicate. Since the length of estrous in goats may vary from 21 to 23 days, animals with a stable and consistent cycle length were used in our study. Based on cycle predictions, surgical sampling was performed two to five days before estrous (Day 0 was the predicted nadir of P_4_) to obtain ovaries with MFs. Once estrous at Day 0 was confirmed, CH tissues were collected at Day 0–4 and CL tissues were collected at Day 4–12 of the cycle.

### Sampling

Prior to the surgery, a physical examination was performed, and food and water were withheld for 24 h. Each goat received flunixin meglumine (2 mg/kg, IM), glycopyrrolate (0.01 mg/kg, IM) and ceftiofur sodium (2.2 mg/kg, SC) approximately 30–60 min before surgery. Animals were initially anesthetized with zoletil (3 mg/kg, IM) and butorphanol (0.1 mg/kg, IM). Subsequently, anesthesia was maintained with isoflurane in 100% oxygen. Lactated Ringer solution (3–10 mL/kg, IV) was administered. For laparoscopic procedures, three portals were created, as previously described^32^. After sampling the ovary, Flunixin meglumine (2 mg/kg, IM) was administered for postoperative analgesia. Each goat was monitored for 24 h after surgery. 11 ovaries were collected during the experimental period (4 ovaries with CL, 4 with MF, and 3 with CH or very early CL).

### Specimen preparation for ultrastructural analysis

After sampling ovaries, the tissues were immediately immersed in precooled fixative containing 4% (w/v) paraformaldehyde (PFA) and 0.25% (w/v) glutaraldehyde (GA) in 0.1 M sodium phosphate buffer (PB, pH 7.3). The ovarian tissues were then dissected and trimmed into small cubes (< 1 mm^3^ for Cortex, CH, or CL) or pieces (for MF) in the fixative. For morphological observations, the specimens were transferred into 4% (w/v) PFA and 2.5% (w/v) GA in 0.1 M PB (pH 7.3) for overnight fixation at 4 °C. The tissues were further subjected to a standard protocol of post-fixation (1% Osmium tetroxide in 0.1 M PB for 90 min), dehydration, and embedding (Spurr’s medium). Semi-thin sections (500 nm) were cut for light microscopy after staining with toluidine blue O (TBO). Ultra-thin sections were stained with 10% uranyl acetate (UA) in methanol (20 min) and Reynold’s lead citrate (LC, 4 min). The mitochondria were observed using a transmission electron microscope (TEM, FEI Tecnai G2 TF20 Super TWIN) operating at 120 kV.

To examine cytochrome c oxidase (COX) activity, the trimmed tissues were fixed in fixative for 10 min on ice and rinsed with cold 0.1 M PB three times (10 min each). The specimens were stained for 3 h at 37 °C in the staining solution that contained 5 mg 3,3′-diaminobenzidine tetrahydrochloride, 9 ml sodium phosphate buffer (0.05 M, pH7.4), 750 mg sucrose, 20 μg catalase (dissolved in 0.05 M potassium phosphate buffer, pH 7.0), and 10 mg cytochrome c (dissolved in distilled water) at a volume of 10 ml^33^. After staining, specimens were washed with PB for 1 h and subjected to standard post-fixation and resin (Spurr’s) preparations for ultrathin sectioning and TEM observation.

For immune-gold labeling, the small pieces of tissues were fixed in precooled fixative for 10 min and rinsed with cold 0.1 M PB three times (10 min each). The tissues were subjected to a standard protocol of dehydration without post-fixation. The dehydrated samples in ethanol were transferred into the device of freeze-substitution (FS) at − 50 °C, incubated with 0.1% UA in acetone for 8 h, and washed with acetone three times for 1 h each. The specimens were subsequently infiltrated through an ascending gradient of Lowicryl HM20 resin (10%, 20%, 40%, 60%, 80%, and 90%, 8 h for each concentration, and agitated every 2 h at − 50 °C). The chamber of the FS device was warmed up to − 25 °C at 5 °C/h. The solution was replaced with 100% HM20 three times for 24 h each (agitated every 2 h). After the embedding of HM20, the ultraviolet (360 nm) polymerization was performed at − 25 °C for 48 h. The chamber was later warmed up to 20 °C (5 °C /h) and exposed to ultraviolet radiation for another 48 h. 100 nm thick sections of the tissues were prepared from the polymerized blocks and placed on 2 × 0.5 mm nickel slot grids with carbon support for immuno-gold labeling.

To screen the specimen for further structural analysis, at least three blocks with target cells were processed for TEM observations from every ovary (at least one grid was collected for each block). For every grid, one of the ultra-thin sections was selected for observation and all of the available areas were checked. 2D images were collected in the area without obvious artifacts. The blocks with acceptable structural preservations were processed and cut again for electron tomography or immuno-gold labeling.

For electron tomography, serial sections (200 nm) through the target cells were obtained^34^. Double-tilt electron tomography was performed with a FEI Tecnai TEM operating at 200 kV. The mitochondrial volume was reconstructed, combined and joined with eTomo, while the segmentation and quantitative analysis of mitochondrial 3D structures were performed with Amira.

### Immune-gold labeling

Thin sections placed on nickel grids were blocked with 5% BSA in PBS for 20 min and incubated with the mouse anti-ATP5A (500x, ab14748, Abcam) antibodies in incubation buffer (1% BSA in PBS) for 2 h. Grids were subsequently washed with incubation buffer three times (10 min each). Secondary antibodies, goat anti-mouse IgG (EM.GMHL15, BB International) with 15 nm gold conjugations at 20-fold dilution were further applied and incubated for 1 h. After washing with PBS, the immune-complexes were fixed with 1% glutaraldehyde in PBS for 5 min and washed three times with distilled water (10 min each). The specimens were inspected by TEM operating at 120 kV (FEI Tecnai G2 TF20 Super TWIN).

### Segmentation and statistical analysis

To provide quantitative measurements of mitochondrial structure, the OMM was defined manually throughout serial tomograms using Amira. For each mitochondrion, a central line tree was generated to identify the mean radius and the total length of mitochondrial segments. For the COX stain, positive signals were identified above the same threshold of density in each tomogram. Only the mitochondria with abundant COX signals (most cristae showed COX activity staining) were selected for OMM definition. The distribution of COX activities was assumed to reflect the cristae structure. To obtain the surface and volume of cristae, the COX area was further delimited by the eroded OMM area. For the quantification of immuno-gold labeling, the 2D images containing target cells were acquired at lower magnification. The mitochondrial area with clear boundaries and ATP5A positive signals were manually labeled in ImageJ. The number of gold particles inside each area was counted. For each phase, 6–10 images were collected for quantitative analysis. The quantitative results were subjected to one-way ANOVA, and Dunn’s multiple comparison test was performed to identify significant differences (*P* < 0.05) between groups.

## Supplementary Information

Supplementary Video Legends.

Supplementary Video S1.

Supplementary Video S2.

Supplementary Video S3.

Supplementary Video S4.

Supplementary Video S5.

Supplementary Video S6.

Supplementary Video S7.

## Data Availability

All data generated or analysed during this study are included in this published article (and its Supplementary Information files).
