# Supplementary material for: Dynamic changes in mitochondrial 3D structure during folliculogenesis and luteal formation in the goat large luteal cell lineage
Source: Sci Rep. 2021 Jul 30;11:15564. doi: 10.1038/s41598-021-95161-w (PMC8324910; doi:10.1038/s41598-021-95161-w)
Supplement: Supplementary file 1 — Supplementary Video Legends. [file 41598_2021_95161_MOESM1_ESM.pdf]

Dynamic changes in mitochondrial 3D structure during folliculogenesis and luteal formation in the goat large luteal cell lineage.

Yi-Fan Jiang[1], Pin-Huan Yu[2], Yovita Permata Budi[1], Chih-Hsien Chiu[3], and Chi-Yu Fu[4]*

[1] Graduate Institute of Molecular and Comparative Pathobiology, School of Veterinary Medicine, National Taiwan University, Taipei, Taiwan.

[2] Institute of Veterinary Clinical Science, School of Veterinary Medicine, National Taiwan University, Taipei, Taiwan.

[3] Department of Animal Science and Technology, National Taiwan University, Taipei, Taiwan.

[4] Institute of Cellular and Organismic Biology, Academia Sinica, Taipei, Taiwan.

[*]Corresponding author

Supplementary information

Movie S1. Serial-section electron tomography and segmentation of the mitochondria in a follicle cell from the goat primordial follicle. Mitochondria without physical connections are labeled with different colors.

Movie S2. Serial-section electron tomography and segmentation of the mitochondria in a granulosa cell from the goat mature follicle. Mitochondria without physical connections are labeled with different colors.

Movie S3. Serial-section electron tomography and segmentation of the mitochondria in a developing large luteal cell from the goat corpus hemorrhagicum. Mitochondria without physical connections are labeled with different colors.

Movie S4. Serial-section electron tomography and segmentation of the mitochondria in a large luteal cell from the goat corpus luteum. Mitochondria without physical connections are labeled with different colors.

Movie S5. Serial-section electron tomography and segmentation of the COX-positive area in a granulosa cell from the goat mature follicle. Mitochondria without physical connections are labeled with different colors.

Movie S6. Serial-section electron tomography and segmentation of the COX-positive area in a developing large luteal cell from the goat corpus hemorrhagicum. Mitochondria without physical connections are labeled with different colors.

Movie S7. Serial-section electron tomography and segmentation of the COX-positive area in a large luteal cell from the goat corpus luteum. Mitochondria without physical connections are labeled with different colors.
